# Editorial: Modulation of ferroptosis via metabolic and signaling pathways: therapeutic opportunities across malignancies and degenerative diseases

**DOI:** 10.3389/fphar.2025.1712096

**Published:** 2025-10-06

**Authors:** Alessandra Fiore, Jian Chen, Adriano Martimbianco de Assis

**Affiliations:** ^1^ Department of Neurosciences, Biomedicine and Movement Sciences, Section of Biochemistry, University of Verona, Verona, Italy; ^2^ Institute of Vascular Anomalies, Shanghai TCM-Integrated Hospital, Shanghai University of Traditional Chinese Medicine, Shanghai, China; ^3^ Anhui Province Rural Revitalization Collaborative Technical Service Center, Huangshan University, Huangshan, China; ^4^ Department of Public Health, International College, Krirk University, Bangkok, Thailand; ^5^ Graduate Program in Health and Behaviour, Center of Health Science, Universidade Católica de Pelotas—UCPel, Pelotas, Brazil

**Keywords:** ferroptosis, cell death, oncology, neurodegenrative disease, ischemic injury, fibrosis, natural compounds

## Introduction

Cell biology has undergone a profound shift in its understanding of cell death, which now goes beyond apoptosis and necrosis. Ferroptosis, first described in 2012 (Dixon et al., 2012), is a distinct, regulated form of cell death characterized by its iron dependence and driven by uncontrolled lipid peroxidation within the cell. Unlike apoptosis, ferroptosis lacks features such as chromatin condensation or caspase activation. Rather, its molecular basis lies in a critical imbalance between oxidative damage and antioxidant protection. Excess ferrous iron (Fe2+) acts as a potent catalyst that drives the Fenton reaction, producing a large amount of reactive oxygen species (ROS). The accumulation of these lipid hydroperoxides, which is the defining event of ferroptosis, is normally suppressed by the antioxidant system. One of the main antioxidant systems protecting against the oxidative damage of ferroptosis is the glutathione (GSH) system, in which glutathione peroxidase 4 (GPX4) uses GSH to neutralize hydrogen peroxide (Cardoso et al., 2017; Dringen, 2000). Dysfunction of key components, such as iron overload, inhibition of GPX4, or disruption of the cystine-glutamate transporter, can trigger ferroptosis. Ferroptosis plays a dual role in disease: its pathological induction contributes to degenerative conditions, such as neurodegenerative and cardiovascular disorders, while the heightened susceptibility of cancer cells makes ferroptosis a promising therapeutic target. (Hangauer et al., 2017; Ubellacker et al., 2020; Zhang et al., 2019; Tsoi et al., 2018; Wang et al., 2019; Belaidi and Bush, 2016; Yoritaka et al., 1996; Dumont et al., 2009; Hambright et al., 2017; Linkermann, 2016; Dare et al., 2015; Dabkowski, Williamson, and Hollander, 2008; Lai et al., 2024; Yoshida et al., 2019; Yao et al., 2025; Chen et al., 2023; Chen et al., 2021). This duality makes ferroptosis an especially fascinating and versatile therapeutic target.

The aim of this Research Topic was to capture this dual role by bringing together contributions investigating ferroptosis in different organs and contexts, with an emphasis on translational perspectives. The six studies included here provide a representative and integrative overview: they span oncology, neurology, cardiology, pulmonology, ophthalmology, and orthopedics. Together, they show that ferroptosis is a unifying mechanism spanning diverse biomedical fields.

## Ferroptosis as a therapeutic target in oncology

Cancer cells often reprogram iron metabolism to support their high proliferative needs (Brown et al., 2020). This makes them particularly sensitive to ferroptotic stress. The study by Zhang et al. in this collection demonstrated this principle in diffuse large B-cell lymphoma. The researchers identified the iron-sulfur protein CISD2 as a regulator of ferroptosis and ferritinophagy, showing that its overexpression reduces chemosensitivity, while its inhibition restores drug responsiveness through ferroptosis induction. Importantly, silencing CISD2 not only promoted ferroptosis but also re-sensitized drug-resistant cells to doxorubicin, highlighting ferroptosis as a potential strategy to overcome therapy resistance. This work resonates with a broader trend in oncology: the recognition that ferroptosis can bypass traditional apoptosis-based resistance mechanisms. Alongside GPX4 and system Xc^−^, CISD2 now joins the growing list of ferroptosis regulators that could be exploited therapeutically.

## Ferroptosis as a mediator of degenerative and ischemic injury

While ferroptosis induction may be advantageous in cancer, the opposite is true in acute injuries of the nervous system and the heart, where excessive ferroptosis drives cell loss. In the context of spinal cord injury, Tao et al. showed that tetramethylpyrazine (TMP), a compound extracted from *Ligusticum wallichii*, protects neurons by activating the NRF2–ARE pathway. This was found to reduce lipid peroxidation, iron overload, and mitochondrial dysfunction, thereby improving functional recovery. The findings underscore ferroptosis as a major mechanism of neuronal death after trauma and highlight NRF2 activation as a promising neuroprotective approach. Similarly, in myocardial ischemia-reperfusion injury, another major cause of global morbidity, ferroptosis has emerged as a key pathogenic mechanism. The work by Liu et al. on the enzyme SAT1 showed that its upregulation activates the MAPK/ERK pathway and triggers ferroptotic cell death, worsening ischemic injury. Conversely, inhibiting SAT1 was found to alleviate ferroptosis and protect cardiomyocytes, suggesting a novel cardioprotective strategy. These contributions exemplify how ferroptosis inhibition may complement established neuro- and cardioprotective therapies, offering new hope for conditions where therapeutic options remain limited.

## Ferroptosis in fibrosis, ocular surface disease, and joint degeneration

Beyond cancer and acute injury, ferroptosis also contributes to chronic fibrotic and inflammatory disorders. In pulmonary fibrosis, for example, Chen et al. demonstrated that the traditional herbal formula Gui-Zhi-Fu-Ling-Wan (GFW) alleviates bleomycin-induced lung damage by inhibiting both epithelial-mesenchymal transition and ferroptosis. This finding reinforces the idea that ferroptosis is not merely a bystander in fibrosis but an active driver of tissue remodeling. In ophthalmology, Hou et al. highlighted the role of ferroptosis in dry eye disease. The research groups showed that astaxanthin, a naturally occurring carotenoid, protects corneal epithelial cells by activating the SLC7A11/GPX4 axis and enhancing autophagy. In animal models, this intervention preserved ocular surface integrity and reduced oxidative stress. These data add to the growing recognition that ferroptosis underlies several ocular pathologies, opening new therapeutic perspectives in a field where current treatments remain largely symptomatic. Finally, in osteoarthritis, Gong et al. reported that Paeonol protects chondrocytes from interleukin-1β-induced ferroptosis via the AMPK/Nrf2/GPX4 pathway. By preserving mitochondrial function and reducing oxidative stress, Paeonol not only prevented chondrocyte death but also attenuated inflammatory responses, offering a disease-modifying potential for osteoarthritis management.

Together, these three studies broaden the scope of ferroptosis research to fibrotic, ocular, and musculoskeletal disorders. Their inclusion emphasizes that ferroptosis is not restricted to acute injuries or cancer but plays a pivotal role in chronic, quality-of-life conditions.

## Outlook

The six contributions in this Research Topic collectively convey a crucial message: ferroptosis is a unifying paradigm in the biomedical sciences. It is increasingly evident that ferroptosis lies at the intersection of iron metabolism, oxidative stress, lipid signaling, and inflammation, processes that are central to the pathogenesis of numerous diseases. By spanning multiple organs and pathologies, this Research Topic highlights the translational versatility of ferroptosis. It demonstrates how the same molecular pathway can be targeted in opposite directions, induced in tumors to trigger cell death, or inhibited in degenerative diseases to preserve tissue integrity. This duality underscores ferroptosis as both a vulnerability and a liability, depending on the biological context. Notably, several contributions explored naturally derived compounds and multi-component formulations, such as TMP, Paeonol, astaxanthin and GFW; these studies delineated signaling pathways that both intersect with and operate independently of canonical ferroptotic mechanisms, including mitophagy, NRF2 signaling, autophagy and inflammatory cascades. This convergence of mechanistic cell biology with natural product pharmacology broadens the therapeutic landscape and underscores the relevance of ferroptosis to translational and integrative medicine.

The rapid expansion of ferroptosis research reflects its ability to connect diverse biomedical fields. Moving forward, key challenges include (i) refining robust and clinically actionable biomarkers for ferroptosis and related redox/iron pathways, (ii) identifying and stratifying patient subgroups most likely to benefit from ferroptosis-targeted or ferroptosis-sparing interventions, (iii) deconvoluting and standardizing multi-component preparations to define active constituents and interactions, and (iv) advancing validated preclinical findings toward well-designed clinical trials with harmonized assays and reporting standards. By presenting work across oncology, neurology, cardiology, pulmonology, ophthalmology, and rheumatology, this Research Topic aims to provide a representative and inclusive perspective. In doing so, it not only highlights mechanistic insights but also illustrates the translational promise of ferroptosis as a therapeutic target for various diseases.
